# Knowledge and Attitude Regarding Cutaneous Leishmaniasis Among Adult Population in Tabuk, Saudi Arabia

**DOI:** 10.7759/cureus.52614

**Published:** 2024-01-20

**Authors:** Amirah M Alatawi, Abeer Mohammed M Alanazi, Ibrahim Abdullah S Albalawi, Nour Saleem Mahmoud Abujaser, Yassmeen Hmoud Alblowi, Asmaa Musallam M Alfuhaymani, Omniyyah Mohammed S Alatawi, Tahani Fahad S Alanazi, Danah Mohsen Alqasmi Albalawi, Naif Mohammed M Alanazi

**Affiliations:** 1 Department of Family and Community Medicine, Faculty of Medicine, University of Tabuk, Tabuk, SAU; 2 Department of Pediatrics, Faculty of Medicine, University of Tabuk, Tabuk, SAU; 3 College of Medicine, Tabuk University for Health Science, Tabuk, SAU; 4 Department of Cardiology, King Fahad Specialist Hospital, Tabuk, SAU; 5 Department of Internal Medicine, Faculty of Medicine, University of Tabuk, Tabuk, SAU; 6 Department of Dermatology, Faculty of Medicine, University of Tabuk, Tabuk, SAU

**Keywords:** tabuk city, attitude, knowledge, leishmaniasis, cutaneous

## Abstract

Background

Cutaneous leishmaniasis (CL) is a major health problem in Saudi Arabia. It is caused by the protozoa Leishmania. The vector is female sand flies. In order to develop preventive strategies to reduce the burden of this health problem, public awareness of the disease should be assessed.

Objective

This study aimed to investigate the knowledge and attitude toward CL among the adult population in Tabuk, Saudi Arabia.

Methods

A cross-sectional survey study was conducted on 385 adult participants of Tabuk between April and May 2022. The investigators assembled the survey questions from peer-reviewed articles with some modifications. Google Forms was used to create the online survey. Social media were used to distribute the survey.

Results

The study revealed that hearing about CL was much more common among participants older than 61 years and Saudi males. The most prominent sources of information for the participants on which their answers were based were as follows: families and friends, health care professionals, television or radio, and lastly, newspapers. Subjects older than 61 years also showed more knowledge of the risk factors for contracting CL. However, the participants showed overall poor knowledge of the clinical presentation of the disease, which gender or age group was mostly affected, or how the disease was transmitted. Likewise, their knowledge of sand flies was poor. Most participants did not know if CL was a health problem or not.

Conclusions

The study revealed overall poor awareness of the epidemiological aspects of CL, how it is transmitted, its clinical presentation, and proper management. Our study could help authorities correct the gap in knowledge regarding CL in Tabuk.

## Introduction

Leishmaniases, diseases caused by protozoa of the genus *Leishmania* within the *Trypanosomatidae* family, are transmitted by the bite of infected female sand flies, specifically species such as *Phlebotomus papatasi*. Globally, approximately 1 million new cases of leishmaniasis are recorded annually, highlighting its widespread prevalence [1].

Cutaneous leishmaniasis (CL) was first described in the ninth century; however, the disease remains a major global health problem in the 21st century. This disease has a wide range of manifestations ranging from a simple chronic skin lesion to a serious systemic infection that might be life-threatening depending on the specific species of *Leishmania* and the genetic makeup and immunological status of the patient [2,3].

Leishmaniasis is found in nearly 90 countries, most of which are developing countries, and predominantly affects developing regions, including 13 of the least developed countries [4]. Morsy and Shoura described the first case in Saudi Arabia in 1973 [5]. Since then, Saudi Arabia has been among the top 10 endemic countries and continues to contribute to a large number of globally reported cases annually [4,5].

*Leishmania major* is confirmed as the etiological agent of CL in north-western Saudi Arabia. The molecular detection of *Leishmania* DNA in *P. papatasi* and *P. kazeruni* sand flies supports their role in the transmission of *L. major*. In addition to CL, other forms of the disease, such as visceral leishmaniasis and mucocutaneous leishmaniasis, are also significant health concerns in endemic regions. Visceral leishmaniasis, often caused by *L. donovani* and *L. infantum*, is the most severe form and can be fatal if untreated. Mucocutaneous leishmaniasis, typically associated with *L. braziliensis*, involves both the skin and mucosal surfaces and can lead to severe disfigurement. The diversity of *Leishmania* species and their varying clinical presentations underscore the complexity of leishmaniasis as a global health issue [6]

CL is a prevalent form of the disease. Globally, 0.7-1.2 million new cases of CL occur each year. The number of CL cases that were reported in Saudi Arabia has dramatically dropped because of the well-established leishmaniasis control program (LCP). Therefore, the current situation is that the Kingdom of Saudi Arabia is no longer among the top 10 countries with endemic diseases around the world [7].

The hottest focus for CL in Saudi Arabia is Ha’il. Most of the cases are reported from six regions: Ha’il and Al-Madinah (North West), Al-Qaseem and Riyadh (Central), Al-Hassa (East), and Aseer (South West) [4].

The affected skin areas are the exposed parts of the body, such as extremities and limbs; however, it is largely observed on the face [8,9]. It is characterized by skin lesions around the initial bite site. Each lesion starts with a papule that progresses into a painless ulcer with raised borders. These lesions are usually chronic and heal, leaving scars and disfigurements [10]. Sometimes, cutaneous lesions spread to mucosal surfaces of the mouth, pharynx, or nose, resulting in mucocutaneous leishmaniasis. This form of the disease may develop during or even a long period after the treatment of the disease [11,12].

The diagnosis of leishmaniasis can be initially suspected in patients presenting with non-healing skin lesions, especially if accompanied by a history of travel to endemic regions. For diagnostic confirmation, a range of methods is employed. Direct microscopic detection of amastigotes of *Leishmania* in skin biopsies is a common approach. In addition to skin biopsies, skin smears are also used for microscopic examination, which can be particularly useful in resource-limited settings. The most accurate diagnostic method is polymerase chain reaction (PCR) testing of lesion specimens. PCR is especially advantageous in cases with low parasite burdens, offering high sensitivity and specificity [13].

Regarding CL, Saudi Arabia is still one of the most endemic areas in Western Asia, and the disease remains to be a major health issue in the country. Prevention of the disease in the most affected regions relies largely on residents’ knowledge of the different aspects of the disease and their attitude toward it. In Saudi Arabia, a few community-based studies have been conducted regarding the public’s knowledge and attitude toward CL [4,7,14,15]. Community participation is a cornerstone for the success of prevention and control programs for any disease. Program implementers need to understand the disease-related knowledge and attitude of the community because these are major determinants of community participation. Therefore, the current study was conducted to investigate the knowledge and attitude toward CL among adults in Tabuk, Saudi Arabia.

## Materials and methods

Ethical considerations

The study protocol obtained approval from the Ethics Committee of the University of Tabuk, Tabuk, Saudi Arabia (approval number: UT-214-66-2022). The participants were informed about the study objectives, methodology, risks, and benefits. Subjects who agreed to fill out the questionnaire implied they agreed to participate in the study. The study conserved participants’ privacy, and each subject got a unique identifier code.

Study design, setting, and data

This cross-sectional survey study was conducted targeting the adult population of Tabuk between April and May 2022. The study included both male and female participants aged 18 years and above. While we aimed to reach a broad segment of the city’s adult population, the actual scope of the invitation to participate was based on the capacity of our distribution channels. Specifically, we utilized social media platforms and local community networks to disseminate the survey, estimating that approximately 667,000 adults in Tabuk were effectively reached with our invitation to participate. Participants were selected through a stratified sampling technique, which allowed us to ensure a diverse representation from various districts within the city. We specifically excluded individuals under 18 years of age, those who refused to participate, and those with incomplete survey data.

Study tool and its validation

In designing our survey instrument, we aimed to ensure that it was appropriately tailored to the adult population in Tabuk and its rural areas, referring to residents of the governorates surrounding Tabuk. We developed our questionnaire by drawing questions from peer-reviewed articles that investigated similar topics in different countries and focusing on assessing participants’ knowledge and attitudes toward CL [1,16,17]. Initially crafted in English, the questionnaire underwent linguistic and grammatical revisions by three language experts. Recognizing the importance of making the survey accessible to all participants, including those who are not proficient in English or are non-educated, we subsequently translated the questionnaire into Arabic. This translation was carefully reviewed for accuracy and cultural relevance by native Arabic speakers with expertise in medical terminology.

Furthermore, the questionnaire was evaluated by three medical consultants (two specializing in dermatology and one in family medicine) to ensure its clinical relevance and comprehensiveness. A pilot study involving 15 subjects (who were not included in the final sample) was conducted to verify the clarity and uniform understanding of the questions across participants.

Data collection

Data were collected online by a self-administered survey generated by Google Forms. Twitter, Instagram, WhatsApp, and other platforms were used to spread the survey through the community. Participants were first asked to give consent to participate. The questionnaire collected data about the following: (a) sociodemographics, such as age, gender, level of education, marital status, employment, residence, and nationality; (b) the participant’s knowledge using questions focused on the mode of disease transmission, clinical picture, high-risk groups, commonly targeted body parts, prevention methods, and management; (c) the participant’s attitude toward CL; and (d) the preventive measure they adopted toward the disease.

Sampling and sample size

A simple random sampling technique was used to select participants. The sample size was estimated with an online sample size calculator (Raosoft, http://www.raosoft.com/samplesize.html) using a margin of error of 5% and a confidence interval of 95%, assuming an average response for most of the questions of 50% and depending on an average population size of 667,837 adult population in Tabuk. The required sample size was 384.

Statistical analysis

An Excel spreadsheet was established for the entry of the data. The analyses were carried out with the IBM SPSS Statistics, version 22.0 (IBM Corp., Armonk, NY, USA). Descriptive statistics were carried out to calculate frequencies (N) and percentages (%) for categorical variables and means with standard deviations (SD) for continuous variables. Appropriate statistical tests, including chi-square test, Fisher’s exact test, and independent t-test, were used to analyze the data. Statistical significance was set at p < 0.05.

## Results

The questionnaire was completed and submitted by 385 participants, of whom 67% (258) were females and 33% (127) were males. About 97.1% (374) are Saudi and 2.9% (11) are non-Saudi. Regarding age, five age groups were studied, and they are 18-28, 29-39, 40-50, 51-61, and more than 61 years, representing 58.2% (224), 17.9% (69), 16.9% (65), 6% (23), and 1% (4), respectively. Considering their level of education, they were divided into non-educated, read and write, pre-university, university, and postgraduate, representing 1.3% (5), 2.6% (10), 14% (54), 76.9% (296), and 5.2% (2), respectively. About 29.9% (115) of the participants were employed, while 70.1% (270) were not employed. The majority, 62.1% (239), were single, and 35.6% (137) were married, whereas 2.1% (8) were divorced, and 0.3% (1) were widowed. The majority of participants, 96.6% (372), resided in urban areas, with only 3.4% (13) living in rural areas (Table 1).

**Table 1:** Basic characters of the study group

Character	No %
Age groups	
18-28	224 (58.2%)
29-39	69 (17.9%)
40-50	65 (16.9%)
51-61	23 (6%)
>61	4 (1%)
Gender	
Male	127 (33%)
Female	258 (67%)
Education	
Illiterate	5 (1.3%)
Can read and write	10 (2.6%)
Before university	54 (14%)
University degree	296 (76.9%)
Higher education	20 (5.2%)
Occupation	
Working	115 (29.9%)
Not working	270 (70.1%)
Marital status	
Single	239 (62.1%)
Married	137 (35.6%)
Divorce and widows	9 (2.4%)
Residence	
Urban	372 (96.6%)
Rural	13 (3.4%)
Nationality	
Saudi	374 (97.1%)
Non-Saudi	11 (2.9%)

**Figure 1:**
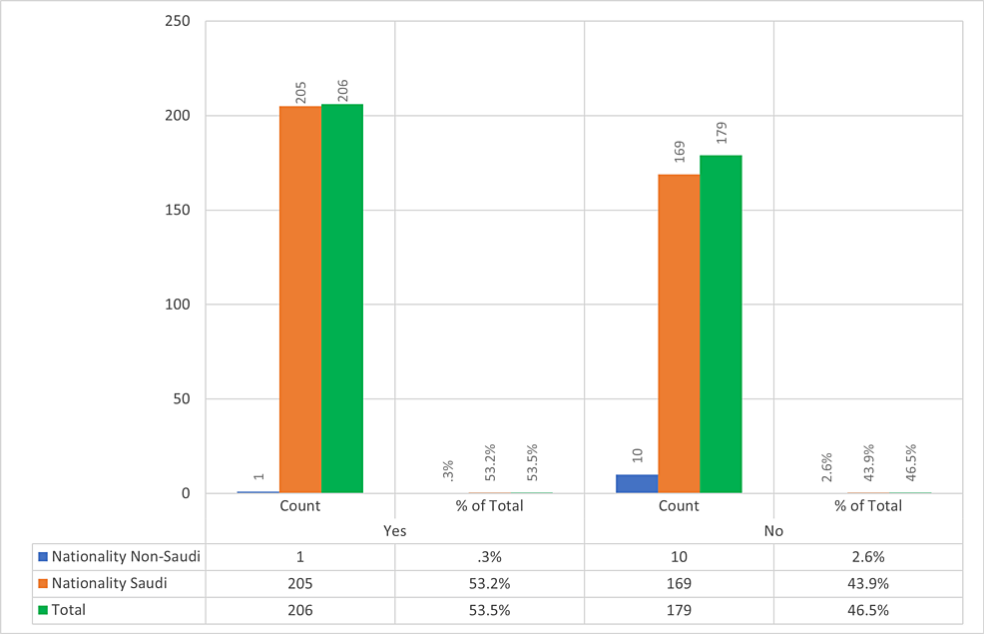
Distribution of CL awareness among study participants, categorized by Saudi and non-Saudi nationalities Awareness of CL by nationality, contrasting responses between Saudi and non-Saudi participants. The blue bar represents the low awareness among non-Saudi nationals (0.3%), while the orange bars show that over half of the Saudi participants (53.2%) are aware of CL. The figure highlights the disparity in CL awareness between the two groups, suggesting a need for focused educational outreach among non-Saudis. CL, cutaneous leishmaniasis

The current study revealed that having heard about CL was notably higher among participants older than 61 years, though it is important to note that only 1% of our study participants fell into this age group, which could introduce a bias in our findings. This age group predominantly consisted of urban Saudi males, primarily at the university or pre-university educational level. The marital and employment status have no effect. Saudi residents heard of CL more than non-Saudis (Figure 1). This could be explained by the fact that CL is an endemic disease in the Kingdom of Saudi Arabia and other certain countries, including Afghanistan, Algeria, and Syria, while non-Saudi residents from non-endemic areas might have never heard of it [4].

**Figure 2:**
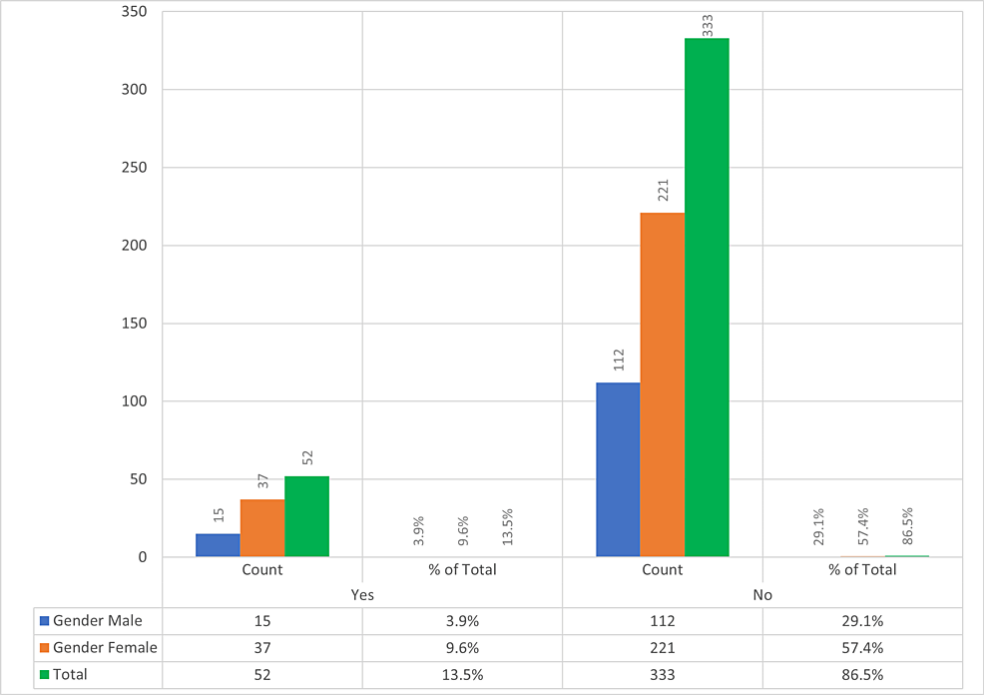
Frequency of encounters with patients diagnosed with CL “Count” refers to the number of survey respondents who have either encountered or not encountered patients diagnosed with CL, categorized by gender. CL, cutaneous leishmaniasis

The health professionals and community or society are the main sources of information about CL in all categories examined. About 333 (86.5%) of the study participants of all age groups and different marital statuses didn’t personally see patients suffering from CL (Figure 2).

The study revealed that a significant portion of subjects across various age groups either acknowledged that factors such as poor housing and sanitary conditions, sleeping outdoors or on the floor, malnutrition, travel to endemic areas, immunodeficiency diseases, and climate changes could predispose individuals to contracting CL, or they indicated a lack of knowledge about these predisposing factors. Upon reanalyzing the data with an adjusted age group combining participants aged 51 and above, it was observed that this broader age category showed more awareness of these predisposing factors. Notably, 50% of participants in this revised age group recognized all the listed factors as predisposing to CL. It was also found that the level of education, employment status, residence, and nationality had no significant influence, as a majority in all categories stated they did not know the correct answer.

Regarding the knowledge of symptoms of CL, our findings indicated that the majority of subjects were either unaware or selected incorrect options, such as painful, disfiguring skin lesions. A closer examination of the responses revealed that a higher proportion of Saudi participants demonstrated awareness of the correct symptoms. Specifically, 43.6% of the Saudi respondents correctly identified the symptoms of CL. In contrast, none of the non-Saudi participants chose the correct answer. This disparity highlights a notable difference in awareness levels between these two groups.

A large portion of subjects of different categories did not know which gender was mostly affected by CL or that CL affects adults more than infants and children. They did not know that the face is the body part mainly affected by CL or that the organism causing CL is a parasite.

In addition, 62.1% of the participants had no knowledge of how CL is transmitted. Their knowledge of sand flies and whether they transmit diseases was variable. Subjects older than 50 years old heard of it more than younger subjects, but that was not statistically significant. Subjects aged between 18 and 28 years old and subjects older than 50 years old showed more knowledge of its ability to transmit diseases. When asked which diseases sand flies transmit, 62.1% of the participants did not know the correct answer (Table 2).

**Table 2:** Knowledge of the participants regarding CL CL, cutaneous leishmaniasis

Knowledge	No %	Mean ± SD
Overall knowledge	159 (41.3%)	31.15 ± 16.49
Risk factor knowledge	137 (35.4%)	3.57 ± 3.33
Symptom knowledge	168 (43.6%)	3.85 ± 3.56
Transmission knowledge	146 (37.9%)	9.60 ± 5.99
Diagnosis and treatment knowledge	199 (51.7%)	14.10 ± 6.44

In the study, approximately 52.5% of the participants indicated that they had not heard about the sand fly, while the remaining 47.5% were aware of it. Only rural residents (38.5%) showed more knowledge of the right breeding place. Sand flies bite more at dusk. Likewise, a large portion of participants did not know that.

In our study, when analyzing the knowledge about the breeding places of sand flies, we found a noticeable difference between rural and urban residents. Specifically, 38.5% of rural residents correctly identified the breeding places of sand flies, compared to only 19.3% of urban residents. This highlights a higher level of awareness among the rural population in this regard. Additionally, regarding the knowledge about the peak biting time of sand flies, which is more at dusk, our data indicated that 63.4% of all participants were unaware of this fact. This gap in knowledge was prevalent across both rural and urban participants.

**Figure 3:**
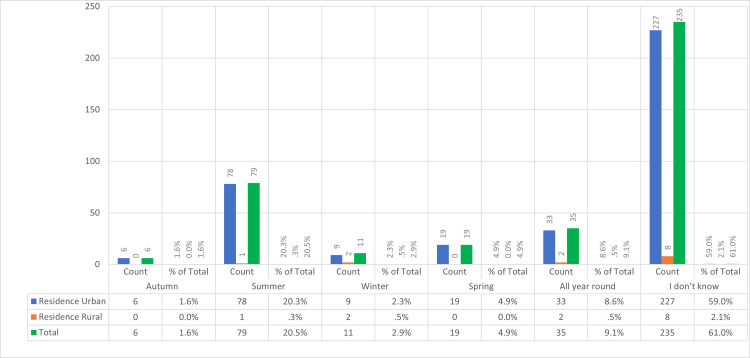
The count of respondents across different residence types (urban and rural) for each peak season of CL CL, cutaneous leishmaniasis

Half of the subjects above 61 years old knew that summer is the peak season for CL. Otherwise, 235 (61%) of the study subjects of different categories stated they did not know (Figure 3).

Among the study participants, those older than 50 years showed a greater awareness of the methods used for sand fly bite prevention compared to other age groups. It was observed that a majority of the other groups, however, indicated a lack of knowledge about these prevention methods.

**Figure 4:**
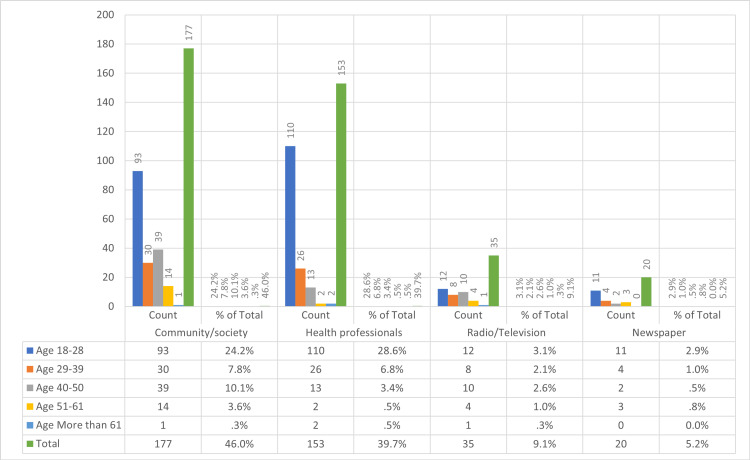
The count of respondents across different age groups for each source of information

About 48.3% of participants of different age groups showed poor knowledge of the management of CL, such as when it should be treated and even the different types of treatment. When asked whether they think CL is a health problem in their area, the majority of participants stated they did not know. In our study, we sought to understand participants’ perceptions of CL as a serious disease. To ensure a consistent understanding, we provided a clear definition of “serious disease” in the survey, describing it as a condition that significantly impacts health and daily functioning. Our findings, based on this defined criterion, suggest that perceptions of CL’s seriousness varied, with responses indicating that it is considered a serious disease or showing a lack of knowledge on this aspect. The sources of information on which the participants based their answers were based were as follows: families and friends, health care professionals, television or radio, and lastly, newspapers (Figure 4).

In our study, a significant majority of participants, 49.4%, were aware that CL is a preventable disease. Regarding the preferred treatment modality, 54% of the participants correctly identified modern drugs as an effective option for treating CL. Furthermore, an interesting observation was that 62.9% of participants indicated that they had employed all known preventive measures against CL. This level of awareness about CL treatment and prevention methods is a positive indicator of public health education effectiveness in the region.

## Discussion

Leishmaniases are vector-borne parasitic diseases caused by at least 20 species of the genus *Leishmania*. The vector is female sand flies. There is a wide range of clinical presentations of leishmaniasis, from self-curing cutaneous lesions to life-threatening visceral disease. CL is usually limited to an ulcer that self-heals over three to 18 months but can also lead to scarring and disfigurement [18].

Although health authorities in the Kingdom of Saudi Arabia continue to exert great efforts, CL is still a major public health issue. It is endemic in Al-Qaseem, Riyadh, Al-Hassa, Aseer, Ha’il, and Al-Madinah. According to Abuzaid et al. [4] regarding Tabuk, 1136 cases were reported from 2006 to 2015.

Making sure that the residents of endemic diseases know the necessary information about the disease is imperative in establishing sound prevention programs. Therefore, this study aimed to assess the knowledge and attitude of adults of Tabuk toward CL.

Our study found that a significant portion of the study population in Tabuk was unaware of CL, indicating a gap in knowledge compared to other regions. For instance, in a study conducted in Central Morocco in the province of Elhajeb in April and May 2019, over half of the participants had heard of CL [19]. Similarly, in a cross-sectional study in Ethiopia in 2019, in two villages of the Ganta-Afeshum district, nearly all participants (99%) were aware of CL [20]. These comparisons highlight the need for enhanced public health education and awareness initiatives about CL in Tabuk.

In our study, awareness of CL was notably higher in the age group over 60 years, with 75% of participants in this category having heard of it. However, it’s important to note that this age group comprised only four participants, which may not be representative of the general population and could indicate a participation bias, given the digital format of our survey. This aligns with findings from Alharazi et al. [21] in Taiz, Yemen, where 500 households were studied. The percentage of participants aged over 40 years with good knowledge about CL was significantly higher than younger participants aged 18-40 years. This could be attributed to increased general knowledge over time, exposure to others with CL, and decreased occurrence of CL due to effective control programs.

Various sources, including health professionals, social media, television, radio, and newspapers, can contribute to public knowledge about CL. In our study, health professionals and the community emerged as the primary sources of information about CL. This observation is similar to that made by Alharazi et al. [21], where a significant proportion of their respondents, 74.5%, reported receiving information about CL through their families, relatives, and friends. Meanwhile, in a study conducted on 400 residents of the Malakand region in Pakistan [22], the knowledge of CL was primarily through teachers and lecturers. Using different sources to address different society categories would be beneficial in increasing awareness of the disease.

When asked whether they personally saw a person suffering from CL, 86.5% of our study participants stated they didn’t. This percentage varied across age groups. Notably, a higher percentage of participants in the older age groups, particularly those over the age of 60, reported having seen someone with CL. This observation aligns with the historical prevalence of the disease, suggesting that older individuals may have had more opportunities to encounter CL cases during times when the disease was more prevalent. Conversely, in the studies of Alharazi et al. [21] in Yemen and El-Mouhdi et al. [19] in Morocco, 76% and 69.4% of the participants said they had seen a patient with CL, respectively. This could be due to the efforts made by the LCP by the Saudi Ministry of Health, which led to a dramatic decrease in the number of reported cases [4].

There are risk factors that increase the possibility of infection, such as poor housing and sanitary conditions in homes, sleeping outside or on the floor, malnutrition, travel to endemic areas, immunodeficiency diseases, and climate changes [23]. Regarding knowledge of these factors, in our study, although the results were variable among categories, many of the participants chose they did not know. In another cross-sectional study conducted on 1824 participants in Al-Hassa in the Kingdom of Saudi Arabia, 40% were able to identify the important risk factors. This could be explained by the fact that Al-Hassa is a hot focus of CL compared to Tabuk [4,15].

CL is presented as a painless, disfiguring skin lesion in the uncovered parts of the body, especially the face [24]. The vast majority of our study participants did not know the correct answer to this question. This was not the case in the study of Amin et al. [15] in the Kingdom of Saudi Arabia, who concluded that the participant’s knowledge of the clinical picture and the part mostly affected was satisfactory.

The majority of our participants had no knowledge that CL mainly affects adult males. Tamiru et al.’s [17] report on 408 participants in Northwest Ethiopia has shown that nearly one-third of them knew that males are more affected than females, but the majority did not know that adults are mainly affected.

Concerning the transmission of CL, 62.1% of participants did not know its method. Moreover, the vast majority of participants did not know the organism causing the infection. The poor knowledge of the mode of transmission of CL is consistent with the results of several earlier studies [15,19,21,22].

The results of our study indicated that awareness of sand flies varied among different demographic groups. Participants residing in the outskirts and less urbanized areas near Tabuk, referred to as “rural residents” in our study, along with those above 61 years old, demonstrated greater awareness of sand flies compared to other participants. This finding aligns with studies by Alharazi et al. [21] and Khan et al. [22], where about 50% of participants could distinguish sand flies from other flies. In contrast, a study by El-Mouhdi et al. [19] reported that more than half of their respondents could not differentiate sand flies from mosquitoes.

The breeding place for sand flies was also a matter of poor knowledge among the participants in our study. Likewise, better knowledge was observed among rural residents. This poor knowledge is consistent with previous studies [21,22]. Moreover, the majority of participants did not know when sand flies bite. However, Irum et al. [25] reported that approximately half of the respondents knew the correct answer. Possible reasons for this difference include geographical and environmental variations, the effectiveness of local public health campaigns, socio-cultural factors, differences in access to information, and variations in study design and population demographics.

In all, 61% of the study participants did not know the peak season for CL. This is in accordance with the results reported by Khan et al. [22] in the Malakand region of Pakistan. On the contrary, 35.2% of the participants in Alharazi et al. [21] study in Yemen knew that summer is the peak season for CL.

In all, 51.7% of participants in our study did not know what diseases sand flies can transmit or how sand fly bites can be prevented. Likewise, the majority of the participants (63.5%) in the Alharazi et al. [21] study did not know if sand flies were able to transmit diseases, but some of the participants thought sand flies are capable of transmitting malaria, and they also showed poor knowledge of the prevention methods. Forty-four percent of the participants in the El-Mouhdi et al. [19] study believed that sand flies are incapable of transmitting diseases, and they thought they could not be avoided. On the contrary, more than half of the respondents in the Khan et al. [22] study knew that sand flies transmit CL. Also, in a study conducted by Saberi et al. [26] on middle- and high-school students in Isfahan, Iran, 97.9% knew that sand fly was the vector even though most of them could not identify it.

In all, 58.4% of the study participants did not know whether CL is a health problem. On the contrary, 78% of the participants in Tesfay et al. [20] study believed that CL is a health problem. Knowing whether CL is a serious disease or not was variable among categories. Other studies also showed a lack of knowledge of the answer to this question and gave variable answers [19,21]. However, more than 60% of the respondents in the study conducted by Khan and colleagues [22] thought that CL could be more serious than malaria.

Meanwhile, 59% of our participants believed CL to be a treatable disease. The belief that CL is a treatable disease was shared by the majority of the participants in other studies [21,22]. However, when asked about its management, the majority of our study participants showed poor knowledge of CL management, when it should be treated, and the different modalities of treatment that can be used, but 54% believed that modern drugs would be the best option for patients. In the Tesfay et al. [20] study in Ethiopia, 44% of the respondents had no idea that modern treatment was developed and available for CL. Moreover, Alharazi et al. [21] reported on harmful practices that the participants thought were useful, such as applying acids on CL lesions. The authors explained this by the deteriorating health system in Yemen as a consequence of the war. In El-Mouhdi et al. [19] study in Morocco, although 44.1% reported that they were treated by health care professionals, traditional choices were still used, e.g., vinegar, bleach, and perfumes.

When the participants in our study were asked whether they had used certain CL prevention measures, the majority chose that they used all of them. This could be general knowledge transferred between people even though the rationale for using these measures is not fully understood. This is consistent with the study of Irum et al. [25] in Pakistan, in which a large portion of the participants chose to take preventive measures using bed nets and insecticides. On the contrary, there was a lack of knowledge and use of preventive measures among participants of other studies [19,21]. In fact, none of the participants in Tesfay et al. [20] study identified bed nets and insecticides as preventive measures for CL.

Our study faced several limitations. The cross-sectional design offers only a snapshot of knowledge and attitudes at a specific point in time. The reliance on self-reported data might introduce recall and social desirability biases. Utilizing online platforms for survey distribution could lead to selection bias, potentially excluding certain demographics. The focus on Tabuk limits the generalizability of our findings to other regions or age groups, as our study represents only 1% of the old age population. Additionally, there is a risk of participants misunderstanding survey questions. Future studies could benefit from diverse data collection methods, a broader demographic focus, longitudinal designs, and qualitative methods. It’s also important to include more participants from rural areas, given the higher disease incidence in these regions. The single-center nature of the study limits its generalizability, and future multi-center research could provide more comprehensive insights. Moreover, not specifying the endemic *Leishmania* species in our study area is a significant limitation that future research should address.

## Conclusions

Overall, our study reflects poor knowledge of CL, its clinical presentation, its vector, and its proper management. There is a need to increase public awareness of the disease in Tabuk to help establish preventive measures and reduce the burden of that endemic disease. This will also encourage early seeking of medical advice to avoid unnecessary complications.
